# Opportunity Screening for Early Detection of Gestational Diabetes: Results from the MERGD Study

**DOI:** 10.3390/jcm14207151

**Published:** 2025-10-10

**Authors:** Manju Mamtani, Kunal Kurhe, Ashwini Patel, Manisha Jaisinghani, Kanchan V. Pipal, Savita Bhargav, Shailendra Mundhada, Prabir Kumar Das, Seema Parvekar, Vaishali Khedikar, Archana B. Patel, Hemant Kulkarni

**Affiliations:** 1 M&H Research, LLC, San Antonio, TX 78249, USA; manju.mamtani@mnhresearch.com; 2 Lata Medical Research Foundation, Nagpur 440022, India; dr.kurhe@gmail.com (K.K.); ashwinipatel1993@gmail.com (A.P.); manishajaisinghani1001@gmail.com (M.J.); pipalkanchan17@gmail.com (K.V.P.); savi_179@yahoo.com (S.B.); prabir_das23@rediffmail.com (P.K.D.); khedikarvaishali@gmail.com (V.K.); dr_apatel@yahoo.com (A.B.P.); 3 Dhruv Pathology and Molecular Diagnostic Laboratory, Nagpur 440010, India; dhruvlabs@gmail.com; 4 Daga Memorial Women’s Hospital, Nagpur 440002, India; seemaparvekar@gmail.com

**Keywords:** gestational diabetes, epidemiology, pregnancy outcomes

## Abstract

**Background:** The definitions and approaches used to diagnose gestational diabetes (GD) are varied. The two-step approach recommended by the American College of Obstetricians and Gynecologists (ACOG) combines the sensitivity of a glucose challenge test (GCT) with the specificity of a 3-hour oral glucose tolerance test (OGTT). We investigated if minor modification of the two-step procedure can provide improved detection of GD by identifying a risk group of pregnant women with high risk of GD. **Methods:** We conducted a prospective cohort study of pregnant women enrolled early during pregnancy and followed till delivery. All participants underwent the ACOG-recommended two-step procedure for GD diagnosis. Based on GCT and OGTT results, the participants were divided into four risk groups (RGs): GCT-negative (RG0), GCT-positive but OGTT normal (RG1), single abnormal value on OGTT or raised HbA1c (RG2) and diagnosed GD (RG3). Baseline evaluation included dietary history (24 hour recall) and physical activity. A series of multivariable logistic regression analyses were conducted to estimate the odds of maternal and fetal outcomes. **Results:** A total of 1041 pregnant women were included in the study, of whom 16 (1.6%) were diagnosed as GD. Our two-step approach identified 48 (4.6%) women as GD, while RG2, RG1 and RG0 comprised 75 (7.2%), 218 (20.9%) and 700 (67.2%), respectively. Compared to RG0, RG2 showed a higher likelihood of antepartum complications [odds ratio and 95% confidence interval 2.38 (1.16–4.15)], any adverse outcome without [2.04 (1.17–3.55)] or with cesarean section [2.09 (1.21–3.61)] and primary cesarean section [1.68 (1.01–2.81)] after adjustment for potential confounders. RG2 was also significantly associated with pregnancy-induced hypertension, meconium-stained amniotic fluid and premature rupture of membranes. **Conclusions:** In the study participants, we identified a subgroup (RG2) at high risk of GD with perinatal outcomes showing profile consistent with that of GD.

## 1. Introduction

Pregnancy induces a wide range of metabolic changes in the mother’s body. Late during pregnancy, there occurs a hypercatabolic state characterized by decreased insulin sensitivity that results in increased levels of maternal glucose and free fatty acid concentrations [1]. While these metabolic changes support fetal growth, there remains a risk of over-catabolism that can result in gestational diabetes (GD). The WHO defines GD as hyperglycemia first detected during pregnancy that does not meet the diagnostic criteria for diabetes mellitus [2]. If not detected early and left untreated, GD can lead to adverse outcomes like fetal macrosomia, fetal hypoglycemia and hyper-insulinemia, prematurity, assisted delivery using cesarean section and preeclampsia [3,4]. Early detection during the asymptomatic phase of GD is thus critical.

The scope and definition of gestational diabetes have suffered due to a lack of uniformity. The diagnostic criteria used to define GD have recently been reviewed by Li-Zhen et al. [5] and include a total of 16 different definitions across the world. In the United States, for example, the American College of Obstetricians and Gynecologists (ACOG) recommends a two-step procedure that combines a 1 h glucose challenge test (GCT) with 50 g of glucose load at 24–28 weeks of gestation followed by a 3 h, 100 g oral glucose tolerance test (OGTT) if needed [6]. On the other hand, in India, the National Guidelines recommend a one-step procedure with a 2 h, 75 g OGTT to diagnose GD [7]. It has been shown that when using the one-step procedure, the prevalence estimates of GD might increase by 1.5 to 3 times higher than those estimated using a two-step procedure [8,9,10,11]. This gain in specificity achieved by a two-step procedure, however, comes at the cost of additional screening visits that may not be feasible in low-resource, low-education settings.

A direct consequence of this lack of uniformity is difficulty in comparing the prevalence of GD across studies. Li et al. [12] demonstrated this in an elegant meta-analysis of 90 published studies of gestational diabetes in India. The GD prevalence estimates were consistently and significantly higher in one-step procedures than in two-step procedures. While the one-step procedures may be associated with false-positive identification of GD, the converse that the two-step procedure may be missing cases of GD is also possible. We hypothesized that if the latter is the case, then the two-step procedure recommendation can be further improved to reduce the false-negative error rate without affecting its specificity.

In this study, we investigated a pregnancy cohort in India with the ACOG-recommended two-step procedure and, using potentially altered cutoffs, identified an additional GD risk group. Our primary research question was as follows: Can the ACOG criteria be adapted to the Indian setting by including cases at a high risk of GD? To answer this research question, we investigated the prevalence of GD using modified cutoff definitions for GD and then tested the association of the GD risk groups with observed rates of adverse maternal and fetal outcomes.

## 2. Materials and Methods

**Study participants:** The Markers of Early Risk-stratification of Gestational Diabetes (MERGD) study (registered with the Clinical Trials Registry—India, CTRI/2018/05/013946) was conducted on all eligible and consenting pregnant women reporting to the Daga Memorial Women’s Hospital, Nagpur. This center is the official enrolment center for the PRIME study [13,14] and the proposed work piggybacked on the PRIME cohort. The study center is a secondary care hospital specializing in obstetric care. Eligibility criteria for inclusion in the MERGD study were consecutive, newly registered pregnant women at the Study Center, with a gestational age at first contact < 20 weeks, no history of type 2 diabetes, and who had provided written, informed consent. All study participants were enrolled between 21 May 2018 and 11 August 2018. Follow-up for all outcomes assessment ended with delivery as the endpoint. Last date of follow-up was 22 February 2019. This study was approved by the Ethics Research Committee of the Daga Memorial Women’s Hospital, Nagpur, India on 10 May 2018.

**Figure 1:**
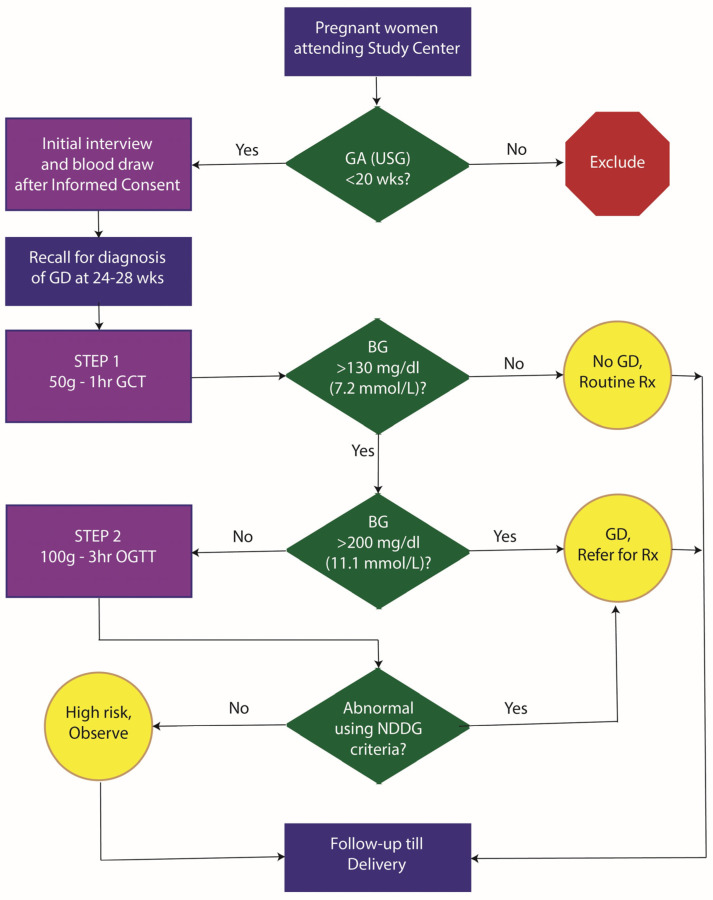
Diagnostic protocol used in the study for the identification of gestational diabetes. GA (USG), ultrasonographic estimate of gestational age; BG, blood glucose; GD, gestational diabetes; NDDG, National Diabetes Data Group.

**Study protocol:** The study protocol for the MERGD study is shown in Figure 1. Eligible pregnant women were first interviewed at the initial study visit. The interview included information on socio-demographics and past obstetric history. After the interview, a blood sample was drawn. This was used for lipid profiling and HbA1c measurement. This initial non-fasting sample was based on a 2 mL draw of blood in EDTA tubes for HbA1c estimation and another 2 mL for lipid profile studies. Furthermore, we stored 10 mL of blood in K3EDTA tubes for subsequent plasma studies and 10 mL of blood in plain tubes for subsequent serum studies and genetic studies. Blood samples were collected in a single prick using Vacutainer technology. Enrolled participants were then invited for a GCT between 24 and 28 weeks of gestation. The GCT was conducted in a non-fasting state and was followed by a detailed dietary history using a 24 h recall method and assessment of physical activity using an investigator-administered instrument. Patients with GCT response ≥ 200 mg/dL were considered as GD. Those with a GCT response between 130 and 200 mg/dL were invited for a 3 hour OGTT with 100 g glucose load. In the approved study protocol, a diagnosis of GD was given according to the National Diabetes Data Group (NDDG) criteria [15], which are any two abnormal values from the following: fasting—≥105 mg/dL, one hour—≥190 mg/dL, two hour—≥165 mg/dL and three hour—≥145 mg/dL. All GD patients received standard-of-care GD treatment till the end of pregnancy. For the GCT and OGTT, we collected 2 mL blood samples in Flouride/Oxalate bottles for glucose measurements. These samples were transported within 6 h of collection to the study laboratory while maintaining a temperature of 4 °C with icepacks. All assays related to this study were conducted at the study laboratory (Dhruv Pathology and Molecular Diagnostic Laboratory, Laxminagar, Nagpur). All the study participants were followed till delivery to measure maternal and fetal outcomes. The outcomes included: maternal morbidity, maternal mortality, still births, prematurity, post-maturity, intrauterine growth retardation, birth weight, gestational age at birth based on USG, macrosomia and early neonatal deaths.

**Diagnostic criteria for gestational diabetes:** To answer our primary research question, we used the ACOG two-step process with the following diagnostic criteria: The first step included a 50 g glucose load, 1 h GCT, in which a cutoff of 130 mg/dL (7.2 mmol/L) was used to decide the need for an OGTT. The second step was carried out in women who showed an abnormal GCT value and included a 100 g glucose load OGTT. The second step used a 100 g glucose load for a 3 h OGTT. Abnormal glucose values were investigated at the time of glucose load (fasting) and then hourly post glucose load. Out of these four blood glucose estimations, the presence of two or more abnormal values was defined as GD. To define abnormal values, we used the Carpenter–Coustan (C&C) criteria [16], as well as the NDDG criteria mentioned above. The C&C criteria used were as follows: fasting—≥95 mg/dL (5.28 mmol/L); 1 h—≥180 mg/dL (10.0 mmol/L); 2 h—≥155 mg/dL; and 3 h—≥140 mg/dL (7.78 mmol/L).

**Dietary assessment:** The assessment of dietary intake was performed at the time of the Glucose Challenge Test (24–28 weeks). Dietary assessment was carried out with the aim of quantifying the macronutrient intake at the time of initial enrolment. This was carried out using a 24 h recall method at the time of first clinic visit. Dietary information was collected in accordance with the USFDA Automated Multi-Pass Method (AMPM) approach [17], with a dedicated Excel^®^-based routine. Dietary information was collected by trained research staff, who administered the structured questionnaire to the participants. Information was collected on timing, food eaten, standardized (using commonly used weight and volume measures) portion sizes and units consumed. This information was then converted to a total daily intake of calories, proteins, fats, carbohydrates, fiber, calcium and iron. For raw foods, the estimations were derived using the information tables from the Nutritive Value of Indian Foods [18]. For cooked foods, the recipe nutritive value was derived from the Nutritionix Database “https://www.nutritionix.com/ (accessed 19 January 2022)”.

**Assessment of physical activity:** Assessment of physical activity was carried out at the time of Glucose Challenge Test using an investigator-administered questionnaire. This was performed using the PPAQ instrument (developed by Dr Lisa Chasen-Taber, University of Massachusetts, Amherst) [19] and adapted to Indian settings [20]. Participants were asked to select the category that best approximated the amount of time spent in 32 activities including household/caregiving, occupational, sports/exercise and inactivity during the current trimester. At the end of the PPAQ, an open-ended section allowed the respondents to add activities not already listed. Excel^®^-based macros were written to estimate the duration and intensity of each activity. These estimates were then used to calculate the metabolic equivalents (METs per week) for each group of activities, as well as the total activity.

**Statistical analyses:** Descriptive statistics included mean (standard deviation) for continuous variables and numbers (%) for categorical variables. The statistical significance of continuous variables across the GD risk groups was tested using the Kruskal–Wallis test while that for categorical variables was tested using Pearson’s chi-square test. Association of the GD risk groups with maternal and fetal outcomes was tested using logistic regression models adjusted for age, BMI, total cholesterol, triglycerides, HDL, LDL, VLDL, systolic and diastolic blood pressure, heart rate, total calories, fats, proteins, carbohydrates, fiber, iron, calcium and water intake and total weekly METs. To quantify the potential, unmeasured, residual confounding, we used the results from the multivariable logistic regression analyses and estimated the e-value [21] for each outcome studied. Statistical significance was tested at a type I error rate of 0.05. All statistical analyses were conducted using the Stata 14 statistical package (Stata Corp, College Station, TX, USA).

## 3. Results

**Study participants and GD risk groups:** A total of 2121 pregnant women who reported to the study center during the study period were screened for eligibility. From this pool, we enrolled 1041 eligible pregnant women who reported for their first antenatal visit before 20 weeks of gestation. The mean (SD) ultrasonographically estimated gestational age at enrollment was 12.65 (3.58) weeks. The age of the participants ranged from 19 y to 40 y with a mean (SD) of 25.42 (4.03) years. Of the enrolled participants, 479 (46%) were nulliparous.

**Figure 2:**
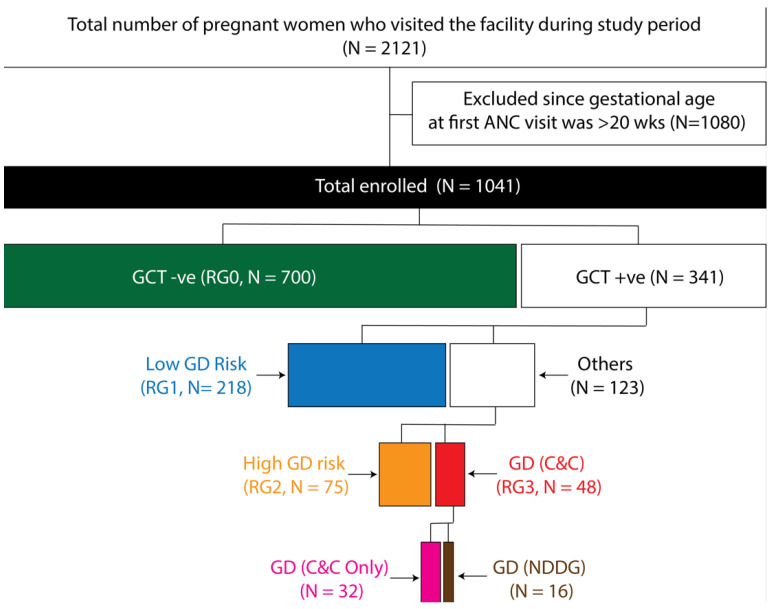
Gestational diabetes risk groups: composition and numbers. From the entire pool of 2121 women who reported to the study center during the study, we excluded a total of 1080 women whose gestational age was >20 weeks at the time of first ANC visit. Of the 1041 pregnant women enrolled into the study, 700 were negative according to the glucose challenge test (GCT)—these patients made up RG0 (green bar). Of the 341 GCT-positive women, 218 had normal OGTT and HbA1c values and made up RG1 (blue bar). Of the remaining 123 women, 75 had either a single abnormal value (according to Carpenter–Coustan criteria) during OGTT or an HbA1c ≥ 6.5% and formed RG2 (orange bar). RG3 (red bar) comprised 48 women, of whom 16 were diagnosed as GD using the NDDG criteria (brown bar).

Based on the results of the GCT, OGTT and HbA1c we created a total of four GD risk groups, as shown in Figure 2. The lowest risk of GD was attributed to the GCT-negative women (denoted as RG0), while the GCT-positive women were further divided into the three remaining risk groups. Women who were GCT positive but had all normal values in OGTT and normal HbA1c (<6.5%) were classified as RG1; women with a GCT-positive result followed by a single abnormal value on OGTT or those with increased HbA1c values were classified as RG2 and those who fulfilled the ACOG to-step procedure with C&C criteria were classified as RG3. Only 16 women were positive for GD using the NGGD criteria and were a subgroup within RG3.

We found (Figure 2) that 67.2%, 20.9%, 7.2% and 4.6% of the study participants belonged to RG0, RG1, RG2 and RG3, respectively. RG2 comprised 50 women who had only a single abnormal value on the OGTT and 25 women who had HbA1c values ≥6.5%. Of the women who had only a single abnormal value on the OGTT, 28 (56.0%) had isolated impaired fasting glucose.

**Baseline characteristics by GD risk groups:** Table 1 details the baseline characteristics of the study participants based on their membership of the GD risk groups. We found that the mean maternal age at enrolment was significantly higher (by approximately 2 years) in RG3 compared to the remaining groups (*p* = 0.0001). Also, the proportion of women with a family income <INR 100,000 per annum was higher in RG3 (*p* = 0.0228). Of note, the body mass index (BMI) showed a steady increase across the GD risk groups such that the mean BMI of RG0 was 21.31 kg/m^2^ while that of RG3 was 23.66 kg/m^2^ (Kruskal–Wallis *p* = 0.0010). Concordantly, the proportion of women with obesity (defined using an Asia-specific cutoff [22] of 27.5 kg/m^2^) was lowest in RG0 (6.4%) and highest in RG3 (22.9%, *p* = 0.0005). Also, both mean systolic and mean diastolic blood pressures were significantly higher in RG3 compared to RG0, with intermediate values in RG1 and RG2 (*p* = 4.4 × 10^−6^ and *p* = 3.4 × 10^−8^, respectively). Finally, the blood lipid profile showed that women in RG3 had significantly higher serum triglycerides (TGs) and serum very low-density lipoprotein (VLDL) concentration (*p* = 0.0001 for both assays). Except for these differences, the participants across GD risk groups were comparable with respect to gestational age at enrollment, obstetric history, proportion of singleton pregnancies, maternal education, caste and religion.

**Lifestyle factors by GD groups:** The results of these analyses are shown in Table 2. The dietary characteristics showed that the total calories, proteins, carbohydrates, fats, calcium and water consumed per day were comparable across the GD risk groups. Interestingly, dietary iron intake and total dietary fiber intake were marginally higher in RG3 compared to the remaining GD risk groups. On the other hand, the metabolic equivalents (METs) expended per week in total and in various subcategories were comparable across the GD risk groups. These observations indicated that at the time of administration of the GCT, the dietary and physical activity characteristics of the study participants did not significantly differ across the GD risk groups.

**Association of GD risk groups with maternal and fetal outcomes:** Supplementary Table S1 shows the distribution of the observed, unadjusted maternal and fetal outcomes across the GD risk groups. There was a significantly high proportion (>61%) of women in RG2 and RG3 with antepartum maternal complications other than GDM. Also, the proportion of women in RG2 with intrapartum maternal outcomes was higher compared to the rest of the GD groups. However, the postpartum maternal adverse outcomes were relatively rare (<1.5%) and comparable across GD risk groups. Indeed, the proportion of women with high-risk pregnancy (Supplementary Note S1) was 26.4% in RG0, 42.7% in RG2 and 43.8% in RG3 (*p* = 0.0008). Together, these results indicated an increased risk of antepartum and intrapartum maternal adverse outcomes in RG2 and RG3. With regards to the fetal outcomes, prematurity was most common in RG3 (27.1%) but relatively uncommon in RG0 and RG2 (13.9% and 14.7%, respectively). Interestingly, the proportion of fetuses with high birth weight was 16.3% in RG2 and RG3 compared to that in RG0 and RG1 (10.3%, *p* = 0.05). However, the proportion of post mature births was comparable across the GD risk groups. Of the most observed fetal outcomes, meconium-stained amniotic fluid (MSAF, 10.7%), premature rupture of membranes (PROM, 8%) and fetal distress (10.7%) were higher in RG2 compared to all other risk groups. Detailed description of all fetal and maternal outcomes observed is given in Supplementary Table S2. It is noteworthy that the proportion of women in RG2 and RG3 who had coexisting pregnancy-induced hypertension (PIH) was higher than that in RG0 and RG1 (21.1% versus 15.5%).

**Figure 3:**
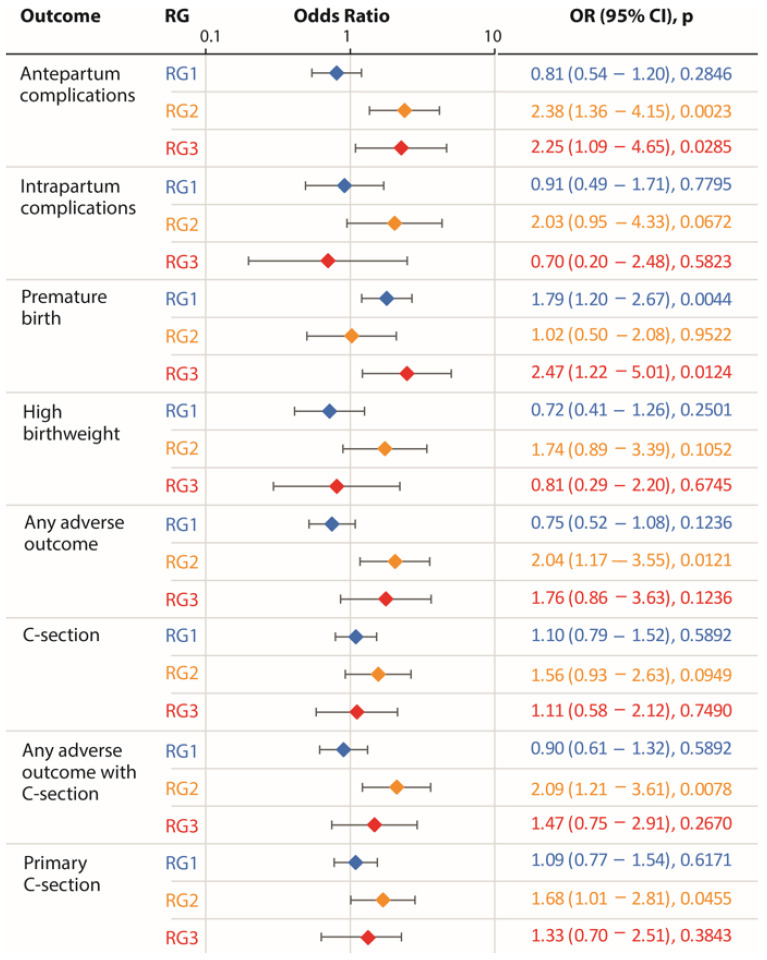
Adjusted odds ratio for study outcomes associated with the GD risk groups. All logistic models are adjusted for age, BMI, total cholesterol, triglycerides, HDL, LDL, VLDL, systolic and diastolic blood pressure, total calories, fats, proteins, carbohydrates, fiber, iron, calcium and water intake and total weekly METs. Diamonds show point estimates, and the error bars show 95% confidence intervals.

The results of adjusted logistic regression models for each outcome of interest are shown in Figure 3. We found that compared to the reference group of GCT-negative women, RG2 was consistently and significantly associated with a higher risk of antepartum complications (*p* = 0.002), a composite outcome indicating presence of any adverse outcome (*p* = 0.012) and any adverse outcome including cesarean section (*p* = 0.008) and primary cesarean section (*p* = 0.46). Furthermore, RG2 showed a marginally significant association with a high risk of intra-partum complications, high birth weight and cesarean section (*p* = 0.067, 0.106 and 0.095, respectively). Interestingly, RG3 showed a significant association with only two outcomes, antepartum complications (*p* = 0.029) and premature births (*p* = 0.012). In total, these observations demonstrated a significantly increased risk of adverse outcomes associated with RG2 group. Using these results, we estimated the e-values for the outcomes with which a significant association of GD risk groups was observed (antepartum complications, any adverse outcome, any adverse outcome with cesarean section and primary cesarean section). The e-values for these outcomes were 2.52, 2.29, 2.22 and 1.88, respectively.

## 4. Discussion

In this study, based on a single abnormal value in the OGTT or raised HbA1c concentration we identified a subgroup of pregnant women who were not diagnosed as GD but were at a high risk of GD (RG2). Since these women were not diagnosed as GD, they received routine antenatal care and consequently were associated with a higher risk of maternal and fetal adverse outcomes. If the diagnostic criteria in the 3 h OGTT were based on a single value (instead of two abnormal values as recommended in the C&C criteria), then our study could have identified a higher number of potential GD cases. Of the 75 women in RG2, 33 (42%) had a high HbA1c at 24–28 weeks of gestation without a clear history of type 2 diabetes diagnosis or treatment previously. Since HbA1c reflects overall glycemia levels over the previous three months, it is possible, in part, that the raised HbA1c levels were related to dysglycemia during the current pregnancy. For this reason, we included HbA1c as an additional indicator of GD in our study. Of note, RG3 comprised only 48 women, indicating that if one were to use the rigid two-step criteria for diagnosis, our cohort would have reported a low prevalence of GD (4.61%). Together, we identified a subset of pregnant women at an increased risk of GD by more accommodative and relaxed criteria.

The two-step diagnostic protocol for GD attempts to combine the high sensitivity of the GCT (using a low threshold of 130 mg/dL) with the high specificity of the OGTT. However, the need for at least two abnormal values on the OGTT implicitly eliminates the importance of isolated impaired fasting glucose in the pathogenesis of GD. Ryan et al. [23] and Kaul et al. [24] have demonstrated through large-scale population studies that impaired fasting glucose is a stronger predictor of large-for-gestational-age infants and hypertension during pregnancy compared to women with impaired glucose tolerance only. These observations corroborate the findings from an elegant, large meta-analysis [25] that demonstrated the importance of a single abnormal value on the OGTT as a significant predictor of poor pregnancy outcomes. Our results agree with this contention and demonstrate that the yield of the GCT is likely to be enriched if the OGTT uses a relaxed criterion of a single abnormal value as diagnostic of GD.

In this context, it is noteworthy that adoption of the one-step recommendations given by the International Association of Diabetes in Pregnancy Study Group (IADPSG, single OGTT with 75 g glucose load, three glucose measurements and a single abnormal value as indicative of GD) has been reported to increase the prevalence of GD owing to the relaxed criteria. However, large randomized controlled trials and systematic reviews [26,27,28,29] have shown that the higher yield of GD in one-step screening was not associated with adverse pregnancy outcomes. In contrast, our identification of RG2 clearly demonstrates a higher likelihood of adverse pregnancy outcomes, thereby reducing the likelihood of overdiagnosis of GD. We therefore believe that the approach of using a single abnormal value in the two-step procedure represents a trade-off between the overdiagnoses implicit in the IADPSG method compared to the risk of missed GD using the ACOG-recommended two-step procedure.

The pattern of association observed in our study indicated that compared to the GCT-negative group (RG0), women in RG2 were at a higher risk of antepartum complications, primary cesarean section, any adverse outcome and any adverse outcome with cesarean section. Also, from among the fetal outcomes, we observed that MSAF and PROM were most common in RG2 compared to other GD groups. These observations are in line with the known risks associated with untreated gestational diabetes [30,31,32,33,34]. Further, the proportion of women with PIH in RG2 was higher than that in RG0 and Rg1. Together, these results indicate that the pattern of perinatal outcomes observed in RG2 mimicked that known to be associated with untreated gestational diabetes. Of note, RG3 was significantly associated with an increased risk of antepartum complications and premature births. While the association of RG3 with antepartum complications is in line with expectations, the association with premature births can be explained by the corroborating observations that GD cases tend to be treated with early induction, especially if there are coexisting complications like PIH [35,36,37,38]. Consistently, within RG3, those patients who were diagnosed as GD using the NDDG criteria had higher rates of adverse events, primary cesarean section, antepartum complications and premature births compared to the rest of RG3 (Supplementary Figure S1). Contrasting the pattern of association of RG3 and RG2 with the perinatal outcomes in this study supports the hypothesis that RG2 comprised women in whom early identification of GD could have been possible.

Our study has some limitations. First, observational data—like the data studied here—cannot be used to infer causality. All the observations made here should therefore be considered evidence of association and not causality. A causal association between RG2 and adverse perinatal outcomes needs to be evaluated in well-designed and controlled settings. Second, the intricate interplay of coexisting conditions and their treatments on perinatal outcomes can confound the interpretations of associations. We conducted logistic regression analyses that adjusted for the potential confounders; however, the potential for unmeasured residual confounding cannot be refuted. The estimated e-values indicated that the unmeasured confounder would need to have an association strength of approximately two or more to be able to influence and confound the observed association. The covariates listed in Table 1, however, indicate that the possibility of missing out on such a strongly associated confounder is low. Third, we used the ACOG-recommended two-step procedure with a 3 h OGTT based on 100 g glucose load. The national practice in India is to use a 2 h OGTT with 75 g glucose load [7]. Therefore, a direct comparison of the incidence of GD in our study sample by the two-step and one-step procedures is not possible.

## 5. Conclusions

Notwithstanding these limitations, our results point to a potential for early diagnosis of gestational diabetes using the ACOG-recommended procedure and the C&C diagnostic criteria. In the ongoing debate [11,39,40,41,42,43,44] about the generally accepted cost-effectiveness superiority of the two-step versus one-step procedure for GD diagnosis, it needs to be highlighted that the two-step process can yield more meaningful results by simple modification of the diagnostic criteria. To that end, this study demonstrates the potential to improve early diagnosis of GD by including a single abnormal value and HbA1c concentration at the time of the OGTT. Larger, controlled studies are needed to definitively address the comparative effectiveness of various GD diagnostic approaches.

## Figures and Tables

**Table 1:** Baseline characteristics of the study participants based on membership of the GD risk group, MERGD 2018.

Characteristic	GD Risk Group				*p*
	RG0	RG1	RG2	RG3	
	(n = 700)	(n = 218)	(n = 75)	(n = 48)	
Enrollment characteristics					
Maternal age at enrollment	25.08 (0.15)	25.85 (0.27)	25.91 (0.56)	27.56 (0.58)	0.0001
Gestational age at enrollment	16.25 (0.11)	16.17 (0.19)	15.47 (0.38)	15.76 (0.46)	0.2204
Singleton pregnancy	688 (98.29)	212 (97.25)	75 (100.00)	47 (97.92)	0.4742
Demographics					
Maternal education					0.1490
Never schooled/kindergarten only	5 (0.72)	2 (0.92)	2 (2.67)	2 (4.17)	
Class 1–8	109 (15.62)	39 (17.89)	16 (21.33)	4 (8.33)	
Class 9–10	235 (33.67)	78 (35.78)	24 (32.00)	13 (27.08)	
Class 11–12	194 (27.79)	54 (24.77)	20 (26.67)	17 (35.42)	
College 1–3 years	106 (15.19)	35 (16.06)	11 (14.67)	6 (12.50)	
College > 3 years	35 (5.01)	8 (3.67)	2 (2.67)	6 (12.50)	
Degree/Masters	14 (2.01)	2 (0.92)	0 (0.00)	0 (0.00)	
Family income					0.0228
INR < 100,000 per annum	287 (41.41)	84 (39.25)	31 (41.47)	23 (48.94)	
INR 100,000–<200,000 per annum	312 (45.02)	102 (47.66)	29 (39.73)	12 (25.33)	
INR 200,000–<300,000 per annum	68 (9.81)	26 (12.15)	8 (10.96)	8 (17.02)	
INR 300,000–<400,000 per annum	20 (2.89)	1 (0.47)	3 (4.11)	2 (4.26)	
INR 400,000–<600,000 per annum	4 (0.58)	0 (0.00)	2 (2.74)	1 (2.13)	
INR 600,000–<1,000,000 per annum	1 (0.14)	1 (0.47)	0 (0.00)	0 (0.00)	
INR ≥ 1,000,000 per annum	1 (0.14)	0 (0.00)	0 (0.00)	1 (2.13)	
Caste					0.0752
Open	205 (29.33)	70 (32.11)	33 (44.00)	22 (45.83)	
Other backward classes	244 (34.91)	78 (35.78)	24 (32.00)	14 (29.17)	
Scheduled caste	166 (23.75)	42 (19.27)	13 (17.33)	4 (8.33)	
Scheduled tribe	33 (4.72)	15 (6.88)	2 (2.67)	4 (8.33)	
Nomadic tribe/Vimukta Jaati	29 (4.15)	8 (3.67)	2 (2.67)	4 (8.33)	
Other	22 (3.15)	5 (2.29)	1 (1.33)	0 (0.00)	
Religion					0.1956
Hindu	427 (61.17)	136 (62.67)	41 (54.67)	26 (54.17)	
Buddhist	123 (17.62)	34 (15.67)	12 (16.00)	4 (8.33)	
Muslim	145 (20.77)	44 (20.28)	22 (29.33)	18 (37.50)	
Sikh	3 (0.43)	1 (0.46)	0 (0.00)	0 (0.00)	
Christian	0 (0.00)	1 (0.46)	0 (0.00)	0 (0.00)	
Other	0 (0.00)	1 (0.46)	0 (0.00)	0 (0.00)	
Obstetric history					
Previous pregnancies					0.2560
0	316 (45.21)	98 (44.95)	43 (57.33)	22 (45.83)	
1	251 (35.91)	67 (30.73)	18 (24.00)	15 (31.25)	
2	105 (15.02)	39 (17.89)	8 (10.67)	10 (20.83)	
3	20 (2.86)	12 (5.50)	4 (5.33)	1 (2.08)	
4	5 (0.72)	1 (0.46)	1 (1.33)	0 (0.00)	
5	2 (0.29)	0 (0.00)	1 (1.33)	0 (0.00)	
6	0 (0.00)	1 (0.46)	0 (0.00)	0 (0.00)	
Previous livebirths					0.6558
0	68 (17.75)	20 (16.67)	7 (21.88)	6 (23.08)	
1	265 (69.19)	84 (70.00)	19 (59.38)	14 (53.85)	
2	44 (11.19)	15 (12.50)	6 (18.75)	6 (23.08)	
3	6 (1.57)	1 (0.83)	0 (0.00)	0 (0.00)	
Previous cesarean section	102 (14.57)	35 (16.06)	12 (16.00)	7 (14.58)	0.9490
Body mass index (kg/m^2^)	21.31 (0.14)	21.51 (0.30)	22.18 (0.45)	23.66 (0.70)	0.0010
Obesity (BMI ≥ 27.5 kg/m^2^)	45 (6.43)	19 (8.72)	8 (10.67)	11 (22.92)	0.0005
Blood pressure					
Systolic (mmHg)	100.63 (0.31)	103.30 (0.55)	102.74 (0.99)	105.70 (1.50)	4.40 × 10^−6^
Diastolic (mmHg)	63.24 (0.23)	65.61 (0.44)	65.58 (0.80)	66.99 (1.07)	3.38 × 10^−8^
Pulse pressure (mmHg)	37.43 (0.22)	37.69 (0.40)	37.75 (0.72)	37.77 (0.93)	0.8629
Mean arterial pressure (mmHg)	75.68 (0.24)	78.17 (0.44)	77.57 (0.75)	80.52 (1.19)	1.48 × 10^−8^
Hypertension	7 (1.01)	7 (3.21)	1 (1.35)	3 (6.25)	0.0133
Blood lipid profile					
Total serum cholesterol (mg/dL)	157.07 (1.21)	162.39 (2.17)	159.72 (3.73)	163.19 (4.27)	0.1133
Serum triglycerides (mg/dL)	104.83 (1.80)	112.13 (2.67)	114.15 (5.84)	125.88 (5.91)	0.0001
Serum high density lipoprotein (mg/dL)	50.90 (0.34)	50.25 (0.59)	48.35 (0.92)	50.77 (1.30)	0.1423
Serum low density lipoprotein (mg/dL)	85.21 (1.06)	89.71 (1.94)	88.54 (3.21)	87.24 (3.78)	0.2076
Serum very low-density lipoprotein (mg/dL)	20.97 (0.36)	22.43 (0.53)	22.83 (1.17)	25.18 (1.18)	0.0001

**Table 2:** Dietary and physical activity characteristics based on gestational diabetes status, MERGD 2018.

Characteristic	GD Risk Group				*p*
	RG0	RG1	RG2	RG3	
	(n = 700)	(n = 218)	(n = 75)	(n = 48)	
Dietary characteristics					
Total calories (Kcal/d)	1510.26 (28.75)	1489.70 (42.63)	1419.60 (69.90)	1560.37 (83.98)	0.4785
Total proteins (g/d)	50.47 (1.29)	53.04 (2.51)	50.00 (4.54)	56.49 (5.29)	0.5188
Total carbohydrates (g/d)	220.91 (3.48)	214.90 (5.39)	203.73 (8.58)	222.68 (11.91)	0.5581
Total fats (g/d)	46.19 (1.50)	45.33 (2.11)	44.02 (3.30)	47.70 (3.28)	0.1557
Total dietary fiber (g/d)	7.14 (0.20)	6.63 (0.29)	6.53 (0.46)	7.81 (0.61)	0.1258
Dietary iron (mg/d)	15.41 (0.31)	13.89 (0.48)	14.25 (0.81)	16.92 (1.07)	0.0136
Dietary calcium (mg/d)	482.24 (10.29)	473.98 (16.71)	485.82 (28.45)	548.71 (50.73)	0.5046
Water intake (L/d)	1.13 (0.02)	1.16 (0.04)	1.11 (0.06)	1.09 (0.06)	0.9137
Physical activity (per week)					
Total METs	59.22 (0.88)	57.42 (1.41)	56.71 (1.84)	56.31 (2.83)	0.5037
Sedentary METs	1.18 (0.07)	1.02 (0.12)	0.84 (0.13)	1.26 (0.24)	0.3199
Light intensity activity METs	49.73 (0.62)	48.44 (1.03)	49.53 (1.52)	48.64 (2.21)	0.7153
Moderate intensity activity METs	8.29 (0.37)	7.95 (0.58)	6.35 (0.52)	6.41 (0.99)	0.5379
Vigorous intensity activity METs	0.02 (0.01)	0.01 (0.01)	0.00 (0.00)	0.00 (0.00)	0.6339
Household/caregiving activity METs	55.65 (0.84)	54.06 (1.38)	53.65 (1.78)	52.67 (2.67)	0.5665
Occupational activity METs	0.04 (0.02)	0.10 (0.05)	0.00 (0.00)	0.00 (0.00)	0.2556
Sports activity METs	1.14 (0.04)	1.11 (0.06)	1.07 (0.10)	1.20 (0.13)	0.9246

## Data Availability

The data that support the findings of this study are not publicly available since restrictions apply to the availability of these data, as outlined in the recommendations of the Ethics Research Committee of the Lata Medical Research Foundation, Nagpur, India. Data are, however, available from the authors upon reasonable request and with permission from the Ethics Research Committee of the Lata Medical Research Foundation, Nagpur, India.

## Supplementary Materials

The following supporting information can be downloaded at https://www.mdpi.com/article/10.3390/jcm14207151/s1, Figure S1: Comparison of the incidence of outcomes within RG3 based on NDDG criteria; Figure S2: Distribution of the antenatal risk score in MERGD cohort; Table S1: Distribution of maternal and fetal outcomes based on gestational diabetes status, MERGD 2018; Table S2: Maternal and fetal outcomes observed in the MERGD study; and Supplementary Note S1: Definition of high-risk pregnancy. References [45,46,47] are cited in Supplementary Materials.

## Abbreviations

The following abbreviations are used in this manuscript:

ACOG	American College of Obstetrics and Gynecology
AMPM	Automated Multi-Pass Method
BMI	Body mass index
C&C	Carpenter–Coustan
GCT	Glucose Challenge Test
GD	Gestational Diabetes
HDL	High density lipoprotein
IADPSG	International Association of Diabetes in Pregnancy Study Group
LDL	Low density lipoprotein
MERGD	Markers of Early Risk-stratification of Gestational Diabetes
MET	Metabolic equivalents
NDDG	National Diabetes Data Group
OGTT	Oral Glucose Tolerance Test
PPAQ	Pregnancy Physical Activity Questionnaire
TG	Triglycerides
VLDL	Very low-density lipoprotein
WHO	World Health Organization
