# Supplementary material for: Opportunity Screening for Early Detection of Gestational Diabetes: Results from the MERGD Study
Source: J Clin Med. 2025 Oct 10;14(20):7151. doi: 10.3390/jcm14207151 (PMC12565406; doi:10.3390/jcm14207151)
Supplement: Supplementary file 1 [file jcm-14-07151-s001.zip › jcm-3860891-supplementary.pdf]

Supplementary Materials

OPPORTUNITY SCREENING FOR EARLY DETECTION OF GESTATIONAL DIABETES: RESULTS FROM THE MERGD STUDY

Supplementary Table S1. Distribution of maternal and fetal outcomes based on gestational diabetes status, MERGD 2018

Outcome	GDM Risk group				P
	RG0	RG1	RG2	RG3	
	(n=700)	(n=218)	(n=75)	(n = 48)	
Maternal					
Antepartum other than GDM	292 (41.71)	81 (37.16)	46 (61.33)	31 (64.58)	$4.6x10^{-5}$
Intrapartum	55 (7.86)	15 (6.88)	11 (14.67)	4 (8.33)	0.1872
Postpartum	9 (1.29)	3 (1.38)	1 (1.33)	0 (0.00)	0.8854
Fetal					
Stillbirth / miscarriage	20 (2.86)	3 (1.38)	1 (1.33)	1 (2.08)	0.5758
Prematurity	97 (13.86)	50 (22.94)	11 (14.67)	13 (27.08)	0.0026
High birth weight	77 (11.00)	18 (8.26)	14 (18.67)	6 (12.50)	0.0995
Postmaturity	23 (3.29)	5 (2.29)	3 (4.00)	1 (2.08)	0.8194
High risk pregnancy*	185 (26.43)	76 (34.86)	32 (42.67)	21 (43.75)	0.0008
Most common					
Pregnancy induced hypertension	109 (15.57)	33 (15.14)	14 (18.67)	12 (25.00)	0.3251
Oligohydramnios	60 (8.57)	18 (8.26)	7 (9.33)	6 (12.50)	0.8079
Polyhydramnios	40 (5.71)	14 (6.42)	5 (6.67)	1 (2.08)	0.6853
Meconium stained amniotic fluid	29 (4.14)	9 (4.13)	8 (10.67)	2 (4.17)	0.0807
Severe anemia	32 (4.57)	7 (3.21)	6 (8.00)	2 (4.17)	0.3929
Premature rupture of membranes	32 (4.57)	2 (0.92)	6 (8.00)	3 (6.25)	0.0243
Fetal distress	18 (2.57)	11 (5.05)	8 (10.67)	3 (6.25)	0.0027
Malpresentation / malposition	27 (3.86)	10 (4.59)	1 (1.33)	2 (4.17)	0.6550
Prolonged labor	26 (3.71)	6 (2.75)	3 (4.00)	2 (4.17)	0.9061

* High risk pregnancy was defined as described in Supplementary Note S1.

Supplementary Table S2. Maternal and fetal outcomes observed in the MERGD study.

Condition Group	Condition	N	%
Placental conditions	Low lying placenta	1	0.10
	Placenta previa	6	0.58
	Placenta accreta	1	0.10
	Placental abruption	2	0.19
Cervical conditions	Cervical cerclage	1	0.10
	Incompetent os	5	0.48
Presentation abnormalities	Borderline pelvis	1	0.10
	Cord prolapse	7	0.67
	Compound presentation	1	0.10
	Cord around neck	2	0.19
	Malpresentation / malposition	40	3.84
Amniotic fluid conditions	Oligohydramnios	91	8.74
	Polyhydramnios	60	5.76
Labor abnormalities	Meconium stained amniotic fluid	48	4.61
	Postpartum hemorrhage	13	1.25
	Prolonged labor	37	3.55
	Premature rupture of membranes	43	4.13
	Vaginal tear	1	0.10
Accompanying conditions	Anemia	47	4.51
	Pregnancy induced hypertension	167	16.04
	Precious pregnancy	1	0.10
	Uterine bleeding	3	0.29
Fetal conditions	IUGR	6	0.58
	Fetal distress	40	3.84
Infections	Dengue	3	0.29
	Acute gastroenteritis	1	0.10
	Enteric fever	6	0.58
	Hepatitis	4	0.38
	Reproductive tract infection	7	0.67
	Tinea	3	0.29
	Viral hemorrhagic fever	1	0.10
	Bleeding per rectum	1	0.10
Other conditions	Hypothyroidism	1	0.10
	Hepatitis	3	0.29
	Hydronephrosis	1	0.10
	Thrombocytopenia	2	0.19
	Varicose vein	1	0.10
	Blood dyscrasias	1	0.10

Supplementary Figure S1. Comparison of the incidence of outcomes within the RG3 group based on NDDG criteria. Blue bars show the observed incidence of the outcome in patients who were diagnosed as GDM using the NDDG criteria (as per the study protocol, n = 16) whereas the orange bars show the corresponding incidence in the remaining RG3 group (that is those who were positive by C-C criteria but did not pass the NDDG criteria, n = 32).

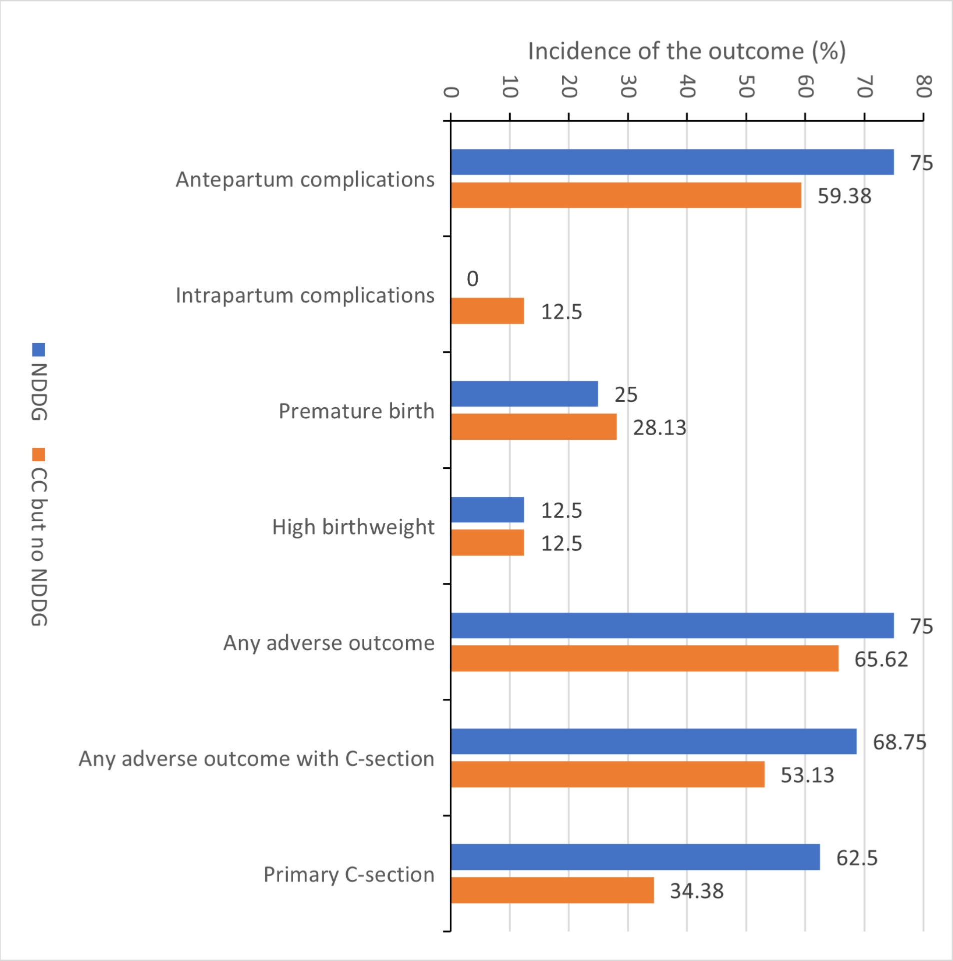

Supplementary Note S1. Definition of High-Risk Pregnancy

We adopted the method and antenatal risk scoring system described by Al-Hindi et al[1] to define high-risk pregnancy in our pregnancy cohort. The risk scoring system included items from three parts: Part A related to prepregnancy characteristics such as maternal age, first visit weight, height, existing comorbidities such as diabetes, hypertension, and chronic kidney disease; Part B included items related to past obstetric history such as neonatal deaths, stillbirth, caesarean section, large for date/small for date, and congenital anomalies; Part C included coexisting problems in the current pregnancy – bleeding, anemia, pregnancy induced hypertension, multiple pregnancies, malpresentations, polyhydramnios or oligohydramnios. The scores for each of these items have been described by Al-Hindi et al[1]. However, to apply to our cohort, we used India-specific cutoffs for maternal age,[2] weight[2] and height[3]. Total antenatal risk score was distributed as shown in Supplementary Figure 2 (below). A total score exceeding 3 was considered as high-risk pregnancy. Using this definition a total of 314 (30.16%) of the participants were classified as high-risk pregnancy.

Supplementary Figure S2. Distribution of the antenatal risk score in MERGD cohort

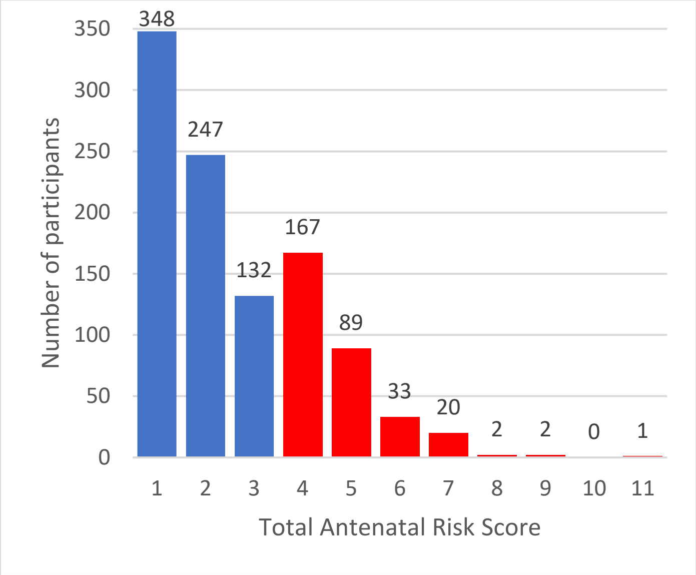

REFERENCES

1. Al-Hindi, M.Y., et al., *Association of Antenatal Risk Score With Maternal and Neonatal Mortality and Morbidity.* Cureus, 2020. **12**(12): p. e12230.
2. Coffey, D., *Prepregnancy body mass and weight gain during pregnancy in India and sub-Saharan Africa.* Proc Natl Acad Sci U S A, 2015. **112**(11): p. 3302-7.
3. Bisai, S., *Maternal height as an independent risk factor for neonatal size among adolescent bengalees in kolkata, India.* Ethiop J Health Sci, 2010. **20**(3): p. 153-8.
